# On the Treatment of Lateral Curvatures of the Spine

**Published:** 1840-01

**Authors:** Jules Guerin


*On the Treatment of Lateral Curvatures of the Spine, by the Subcutaneous Division of the Muscles of the Back and the Vertebral Column. Abstract of a Letter addressed to the Academy of Sciences.* By Jules Guerin, M.D.

I have the honor to communicate to the Academy, the first result of a new operation, which I have already practiced twelve times successfully in subjects affected with lateral curvature of the spine. This operation consists in the section of certain muscles of the back and vertebral column. The muscles which I have at present divided are the trapezius, the rhomboideus, the levator anguli scapulæ, the sacro-lumbalis, longissimus dorsi, and the semi-spinalis.

The greater number of lateral distortions of the spine are the result of active muscular contraction, and their anatomical varieties are the result of this contraction, variously exhibited in the muscles of the spine and back. The active treatment of this class of deformities must, therefore, consist in the subcutaneous section of the muscles, to the shortening of which each deformity is due. The following are some details of the applications which I have made of this mode of treatment:

I have applied it to subjects of both sexes and of different ages: the youngest was thirteen, the oldest thirty-two years old. All the cases were deformed in the second or third degree, with proportionate twisting of the column, and gibbosity. In some a single division of the retracted muscles sufficed; in others, two or three had to be made. In all I obtained, immediately after the operation, a marked degree of straightening of the column; and in a young man of twenty-one, whose deformity had been subjected for eighteen months to mechanical treatment, I obtained by dividing the corresponding longissimus dorsi and semi-spinalis, an immediate removal of the whole deviation. In other subjects I have been able to pursue, with a constant success, the treatment, by mechanical means. In none of the twelve operations have I met with the least accident; there has been no hemorrhage; but little pain; no fever; and in every case but one, immediate union of the wound has taken place, without suppuration. I may add that though delicate, this operation may be performed almost as easily as the similar one in the neck or foot.—*Gazette Medicale. June* 29, 1839.

